# Supplementary material for: Regular-triangle trimer and charge order preserving the Anderson condition in the pyrochlore structure of CsW2O6
Source: Nat Commun. 2020 Jun 19;11:3144. doi: 10.1038/s41467-020-16873-7 (PMC7305186; doi:10.1038/s41467-020-16873-7)
Supplement: Supplementary file 1 — Supplementary Information [file 41467_2020_16873_MOESM1_ESM.pdf]

Supplementary Information

Regular-Triangle Trimer and Charge Order Preserving the Anderson Condition in the Pyrochlore Structure of CsW_2O_6

Okamoto *et al*.

Supplementary Note 1. Structural analyses on the single crystal and powder XRD data

The experimental conditions during the single-crystal XRD measurements, and the crystallographic parameters obtained by the structural analyses of Phase I (250 K) and Phase II (100 K) of CsW_2O_6 are shown in Supplementary Tables 1–4, respectively. Those of $CsW_{1.835}O_6$ are shown in Supplementary Tables 5 and 6. The unit cell of Phase III was determined by structural analyses of the single crystal XRD data, as shown in Supplementary Fig. 1. The temperature dependences of the lattice constants determined by the Rietveld analyses of the powder XRD data of CsW_2O_6 are shown in Supplementary Fig. 2. At the lowest measured temperature of 30 K, the difference between $a/2$ and b and the deviation of β from 90° are 0.04% and 0.08%, respectively, indicating the monoclinic distortion in Phase III is quite small. In contrast to Phase III, Phase II was found to preserve the cubic symmetry. Supplementary Fig. 3 shows the intensities of symmetrically-equivalent reflections of $\underline{12}10$ in single-crystal XRD of Phase II. Intensities of three reflections of $1\underline{12}0$, $01\underline{12}$, and $\underline{12}01$ and those of $\underline{12}10$, $0\underline{12}1$, and $10\underline{12}$ are identical within the uncertainties, respectively, indicating that the Phase II has the $m\bar{3}$ Laue class. Supplementary Figs. 4A and 4B show a peak profile of $\underline{11}97$ reflection (cubic unit cell) at 30, 100, and 300 K and temperature dependence of full width at half maximum of the $\underline{11}97$ reflection, respectively. In the cubic phase, in addition to symmetrically-equivalent reflections of the $\underline{11}97$ reflection, some reflections such as $\underline{13}91$ and their symmetrically-equivalent reflections overlap at the same diffraction angle, but they split when symmetry lowering from cubic occurs. The increase of the peak width in Phase III below 95 K reflects such symmetry lowering. In contrast, the peak width in Phase II is same as that in Phase I, indicating that the Phase II has the cubic symmetry.

Supplementary Table 1. Experimental conditions of the single-crystal XRD experiment of Phase I (250 K) of CsW_2O_6.

Chemical formula	CsW_2O_6
Formula weight	596.604
Temperature (K)	250
Wavelength (Å)	0.35491
Crystal system	Cubic
Space group	$Fd\bar{3}m$
Unit cell dimensions (Å)	a = 10.321023(7)
Volume (Å^3)	1099.431(2)
Z	8
Density (calculated) (g cm^{-3})	7.20871
Crystal size (μm)	60 × 50 × 30
Reflections collected (d > 0.35 Å)	19003
Independent reflections	662
Goodness-of-fit	1.264

Final R indices [$F_0 > 4\sigma(F_0)$]	$R1 = 0.0124$, $wR = 0.0339$
R indices (all data)	$R1 = 0.0155$, $wR = 0.0350$

Supplementary Table 2. Crystallographic parameters for Phase I (250 K) of CsW_2O_6.

	Wyckoff position	x	y	z	U_{eq}
W	16c	0	0	0	0.00687(2)
Cs	8b	3/8	3/8	3/8	0.02232(6)
O	48f	0.06041(10)	3/8	3/8	0.01064(10)

Supplementary Table 3. Experimental conditions of the single-crystal XRD experiment of Phase II (100 K) of CsW_2O_6.

Chemical formula	CsW_2O_6
Formula weight	596.604
Temperature (K)	100
Wavelength (Å)	0.35491
Crystal system	Cubic
Space group	$P2_13$
Unit cell dimensions (Å)	a = 10.319398(6)
Volume (Å^3)	1098.912(2)
Z	8
Density (calculated) (g cm^{-3})	7.21211
Crystal size (μm)	60 × 50 × 30
Reflections collected (d > 0.35 Å)	111710
Independent reflections	8584
Goodness-of-fit	0.912
Final R indices [$F_0 > 4\sigma(F_0)$]	$R1 = 0.0192$, $wR = 0.0373$
R indices (all data)	$R1 = 0.0270$, $wR = 0.0390$
Domain ratio	**a**, **b**, **c** : **a**, **c**, **b** : –**a**, –**b**, –**c** : –**a**, –**c**, –**b** = 0.51(3) : 0.01(3) : 0.48(3) : 0.00(3)

Supplementary Table 4. Crystallographic parameters for Phase II (100 K) of CsW_2O_6.

	Wyckoff position	x	y	z	U_{eq}
W(1)	4a	0.378453(5)	0.378453(5)	0.378453(5)	0.00286(1)
W(2)	12b	0.625217(6)	0.374558(6)	0.628481(6)	0.00356(1)
Cs(1)	4a	0.243565(9)	0.256435(9)	0.743565(9)	0.00925(2)
Cs(2)	4a	0.500231(6)	0.000231(6)	0.499769(6)	0.00914(3)
O(1)	12b	0.56046(12)	0.24962(7)	0.75045(8)	0.01268(18)
O(2)	12b	0.50125(6)	–0.00598(6)	0.81972(9)	0.00539(10)
O(3)	12b	0.50121(6)	0.31034(9)	0.49035(7)	0.00576(9)
O(4)	12b	0.74789(6)	0.25218(6)	0.56776(10)	0.00565(10)

Supplementary Table 5. Experimental conditions of the single-crystal XRD experiment of $CsW_{1.835}O_6$.

Chemical formula	$CsW_{1.835}O_6$
Formula weight	566.3
Temperature (K)	30
Wavelength (Å)	0.38813
Crystal system	Cubic
Space group	$Fd\bar{3}m$
Unit cell dimensions (Å)	a = 10.27190(10)
Volume (Å^3)	1083.808(18)
Z	8
Density (calculated) (g cm^{-3})	6.941
Crystal size (μm)	60 × 50 × 20
Reflections collected (d > 0.35 Å)	22015
Independent reflections	1059
Goodness-of-fit	0.989
Final R indices [$F_0 > 4\sigma(F_0)$]	$R1$ = 0.0211, wR = 0.0415
R indices (all data)	$R1$ = 0.0392, wR = 0.0392

Supplementary Table 6. Crystallographic parameters for $CsW_{1.835}O_6$ (30 K).

	Wyckoff position	x	y	z	occupancy	U_{eq}
W	16c	0	0	0	0.9177(16)	0.00687(2)
Cs	8b	3/8	3/8	3/8	1	0.00736(3)
O	48f	0.06132(10)	3/8	3/8	1	0.01064(10)

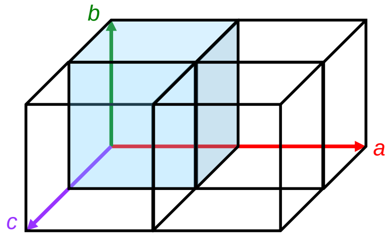

Supplementary Figure 1. A unit cell of Phase III determined by the structural analyses of the single crystal XRD data. The unit cell of Phase III is 2 × 1 × 2 of that of Phase II indicated as the blue shaded region.

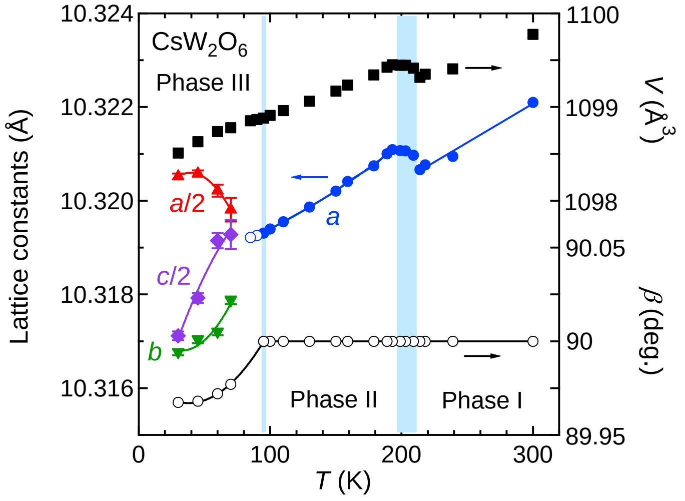

Supplementary Figure 2. Temperature dependence of the lattice constants determined by the Rietveld analyses of the powder XRD data. The lattice constants at 85 and 90 K were refined using the cubic cell because monoclinic distortion was negligibly small. The error bars indicate the standard deviation.

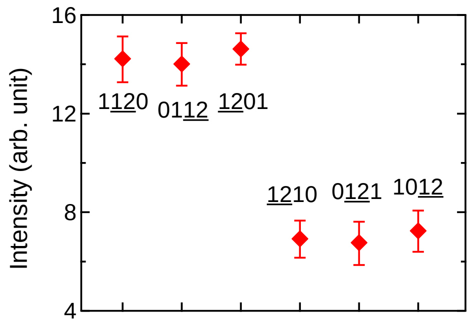

Supplementary Figure 3. Intensities of symmetrically-equivalent reflections of 1210 in single-crystal XRD of Phase II (100 K). The error bars indicate the standard deviation.

Supplementary Note 2. Twinning and space group of Phase III

In Phase III, superlattice reflections were observed at (h/2, k/2, l/2) in the cubic unit cell of Phase I and II. The size of the unit cell and the space group of Phase III were determined by the following procedure. First, since the lattice distortion appears continuously in Phase III, as shown in Supplementary Figs. 2 and 4, the phase transition from Phase II to III was considered to be second order. In this case, the space group of Phase III should be a subgroup of Phase II. According to the group-subgroup relation, $R3$ and $P2_12_12_1$ are the maximal subgroup of $P2_13$. In the $R3$ and its subgroup cases, the observed superlattice reflections could not be indexed. In the case of $P2_12_12_1$, possible unit cell is $a' = 3a$, $b' = 3b$, or $c' = 3c$, but the superlattice reflections appeared at (h/2, k/2, l/2), indicating that this space group is not applicable.

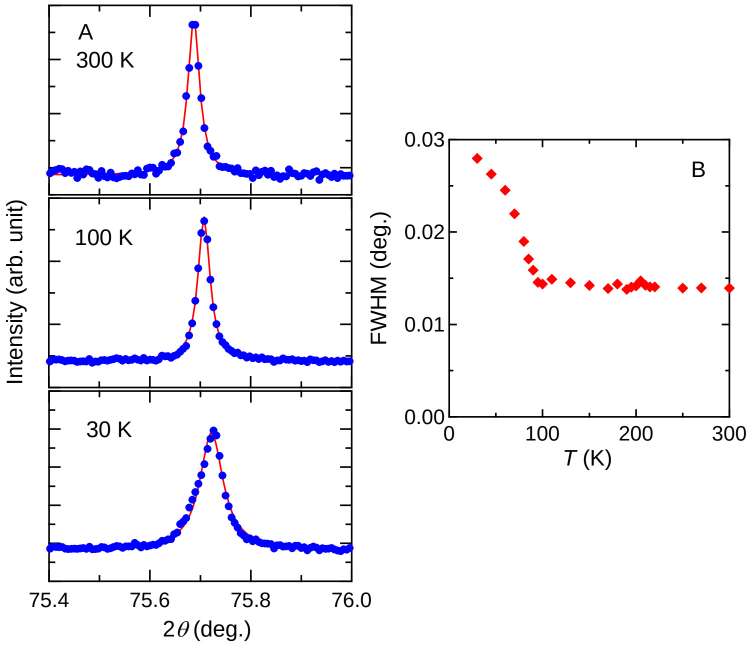

Supplementary Figure 4. (A) Peak profiles of $\underline{11}97$ reflection (cubic unit cell) at 300, 100, and 30 K and (B) temperature dependence of full width at half maximum of the $\underline{11}97$ reflection (lower). The solid curves in (A) show the fitting results to the Lorentzian function.

In contrast, $P2_1$ space group, which is the maximal subgroup of $P2_12_12_1$, can have a unit cell of $a' = 2a$, $b' = b$, and $c' = 2c$. In this case, three domains are formed by the selection of the unique axis of the monoclinic lattice from a, b, and c axes in Phase II. The superlattice reflections of the three domains are partly overlapped. In addition, each of the three domains generates eight domains due to the pseudomerohedral and inversion twins, which give rise to Bragg reflections at the same positions. By considering these 24 domains, all reflections, including superlattice reflections, were indexed. There are other unit cells that reproduce superlattice reflections in this space group, but the above choice minimized the unit cell. Next, in consideration of the overlapping reflections by domains, it was confirmed that the reflections that violate the generation rule of 2_1 spiral symmetry ($0k0$: $k = 2n$) do not exist in the diffraction patterns. Therefore, we determined the unit cell and space group of the Phase III to be monoclinic $P2_1$ with $a' = 2a$, $b' = b$, and $c' = 2c$ shown in Supplementary Fig. 1.

Supplementary Note 3. Structural model in Phase III

At present, it is impossible to determine the atomic position in Phase III, because the unit cell contains a very large number of atoms and reflections of multiple domains overlap each other. In this study, in order to derive the characteristics of the crystal structure of the Phase III, the crystal

structure was considered in the following procedure. First, we indexed the superlattice reflections according to the three domains discussed in the previous section. Then, we extracted the superlattice reflections that do not overlap those of other domains. Comparing the intensities between the three domains, there was no significant difference, so the following analysis assumed that the volume fractions of the three domains were equal. Supplementary Fig. 5A shows the intensities of the superlattice reflections on the *h*0*l* plane (the unit cell of Phase III) in the reciprocal space obtained by this procedure. The superlattice reflections with $h + l = 8n$ have strong intensity, which coincides with the position of diffuse scattering shown in Fig. 4.

The observed superlattice reflections were found to be well reproduced by slight shift and rotation of the W_3 trimers without changing the shape of the trimer. Supplementary Fig. 5B shows a simulated pattern of the intensities of the superlattice reflections, when the trimers were moved to reproduce the pattern of Supplementary Fig. 5A as much as possible. A schematic picture showing the shift and rotation of the trimers is shown in Supplementary Fig. 5C. The simulated pattern is in good agreement with the experimental result, suggesting that the structural change from Phase II to III is not due to a drastic change, such as recombination of the trimers, but to a slight change of the trimer position.

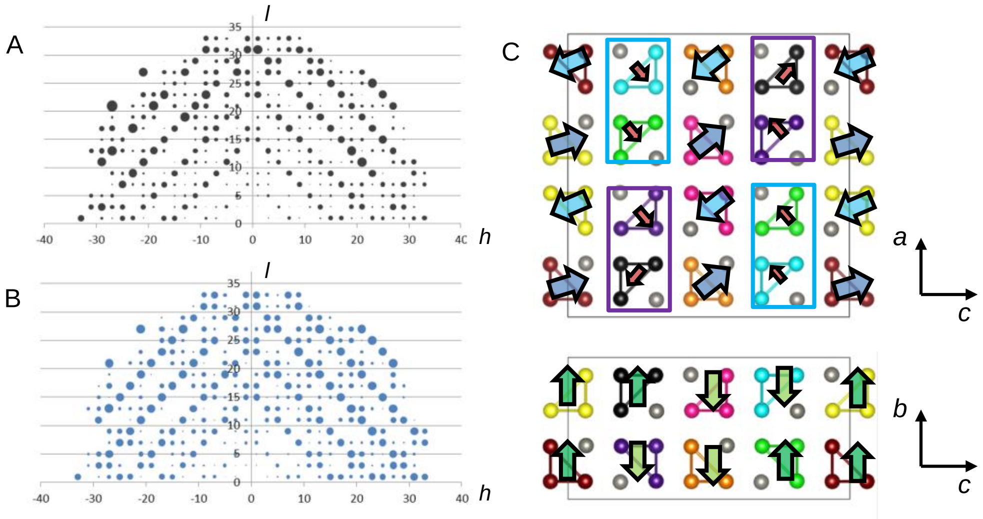

Supplementary Figure 5. (A) Intensities of the superlattice reflections on the *h*0*l* plane extracted from the XRD pattern of Phase III. (B) Intensities of the superlattice reflections on the *h*0*l* plane calculated by using the structural model schematically shown in (C). (C) A schematic picture of the shift and rotation of the W_3 trimers to reproduce the observed intensities shown in (A). The pictures viewed perpendicular to the *ac* and *bc* planes are shown in upper and lower panels, respectively. Spheres and triangles indicate W atoms and W_3 trimers, respectively. The arrows represent the direction and degree of the displacement of trimers.

Supplementary Note 4. Physical properties of W-deficient $CsW_{1.835}O_6$

Supplementary Fig. 6 shows the temperature dependences of the electrical resistivity, ρ, and magnetic susceptibility, χ, of the $CsW_{1.835}O_6$ single crystals. The data of the CsW_2O_6 single crystals are also shown for reference. The ρ value of the $CsW_{1.835}O_6$ single crystal is two orders of magnitude larger than that of CsW_2O_6 at room temperature, and it increases with decreasing temperature. The χ value of the $CsW_{1.835}O_6$ single crystal above 100 K is comparable to that of CsW_2O_6 in Phase II. There are no anomalies in the ρ and χ data of $CsW_{1.835}O_6$, which is in contrast to those of CsW_2O_6. These results indicate that $CsW_{1.835}O_6$ is a nonmagnetic insulator that does not possess *d* electrons, which is consistent with its chemical composition determined by the structural analyses of the single-crystal XRD data. This yields the W valence of 5.995+, indicating that there are almost no 5*d* electrons. χ of $CsW_{1.835}O_6$ shows Curie-Weiss behavior at low temperatures, suggestive of the presence of impurity spins in the sample. A Curie-Weiss fit of $\chi = C/(T - \theta_W) + \chi_0$ to the 2–50 K data yielded C = 0.0118(12) cm^3 K mol^{-1}, θ_W = –9.2(11) K, and χ_0 = –1.04(2) × 10^{-4} cm^3 mol^{-1}. This C value means that 1.6% of the W atoms have an S = 1/2 spin.

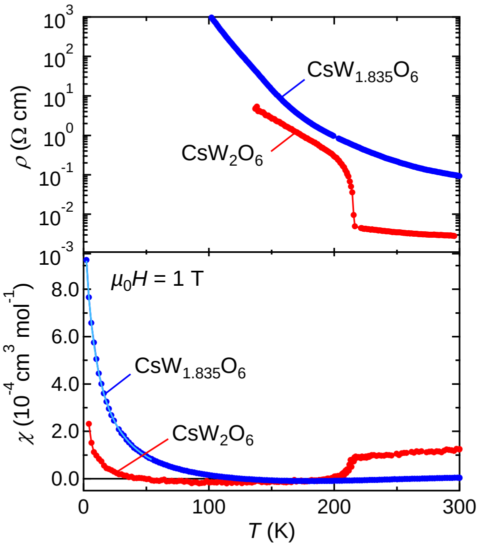

Supplementary Figure 6. Temperature dependences of electrical resistivity (upper) and magnetic susceptibility (lower) of the $CsW_{1.835}O_6$ single crystals. The magnetic susceptibility was measured under a magnetic field of 1 T. The data of CsW_2O_6 are shown for reference. The solid curve in the lower panel shows the result of a Curie-Weiss fit to the $CsW_{1.835}O_6$ data between 2 and 50 K.

Supplementary Note 5. Diffuse scattering in the single-crystal XRD patterns

Diffuse scattering was observed in the single-crystal XRD patterns of CsW_2O_6 and $CsW_{1.835}O_6$. The XRD patterns presented in this section emphasize the presence of diffuse scattering. Since the intensity of diffuse scattering is much lower than those of Bragg reflections, the observed

diffuse scattering has no effect on the crystal-structure refinement. Supplementary Fig. 7A shows XRD patterns of CsW_2O_6 and $CsW_{1.835}O_6$ single crystals measured at 250 and 30 K, respectively, which show diffuse scattering with the same pattern. The intensity of the diffuse scattering for CsW_2O_6 continuously decreases with decreasing temperature, as shown in Supplementary Fig. 7B. No discontinuous change is observed at the phase transitions. On the other hand, that of $CsW_{1.835}O_6$ is almost constant throughout the entire temperature range. Supplementary Fig. 8A is an enlarged view of the XRD pattern of CsW_2O_6 at 250 K. Diffuse scattering has been formed that connects diffraction spots with the same h and l that satisfy the extinction rule of $h + l = 4n$. This extinction rule cannot be explained by the displacement of Cs atoms. The presence of the extinction rule suggests that the diffuse scattering is not caused by imperfections in the crystal. An atomic displacement pattern that can reproduce this extinction rule is shown in Supplementary Fig. 8B, which is characterized by a displacement of W atoms along [101].

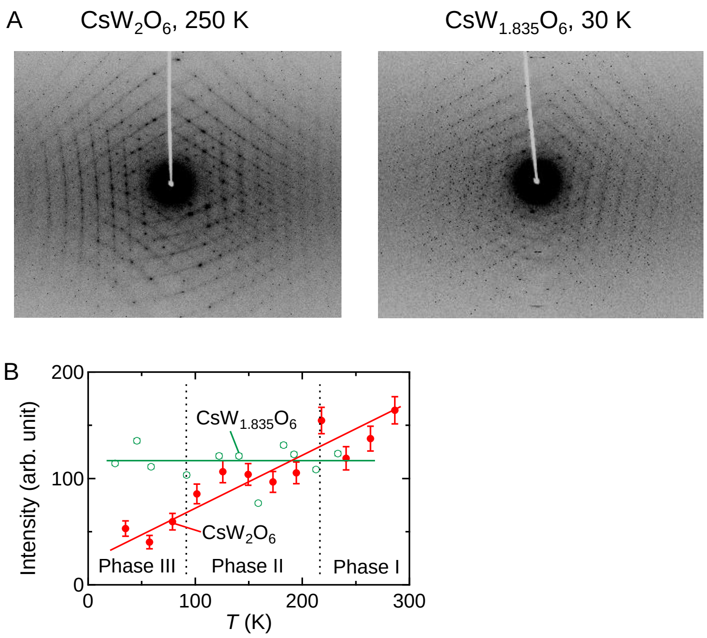

Supplementary Figure 7. (A) Single crystal XRD patterns of CsW_2O_6 of Phase I (left) and $CsW_{1.835}O_6$ (right) measured at 250 and 30 K, respectively. (B) Temperature dependence of the intensity of the diffuse scattering for CsW_2O_6 and $CsW_{1.835}O_6$. The error bars indicate the standard deviation.

Supplementary Note 6. Raman scattering

Raman spectra of (100) surface of CsW_2O_6 are shown in Supplementary Fig. 9. Irreducible representation of each peak is also shown. The scattering peaks are appropriately assigned by the $Fd\bar{3}m$ symmetry, in which the Raman active modes are $A_{1g} + E_g + 4T_{2g}$. One T_{2g} mode is missing, probably due to its weak intensity. The Cs^+ ions behave as a rattling ion in the β-pyrochlore

structure. Vibration of the Cs^+ ions with the T_{2g} mode appears at 55 cm^{-1}, which are comparable to that of KOs_2O_6[14].

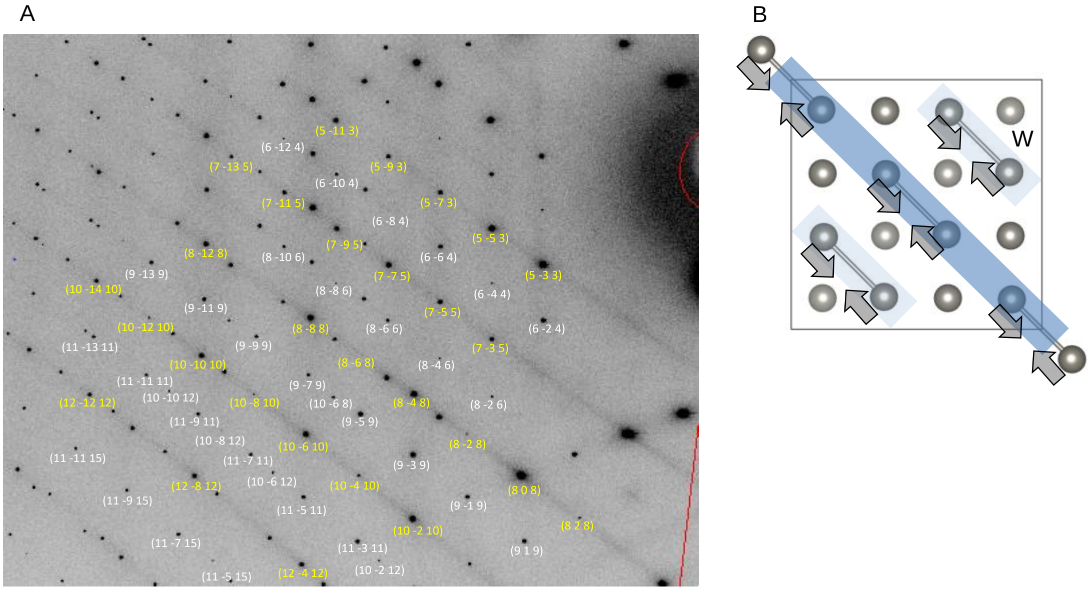

Supplementary Figure 8. (A) A single crystal XRD pattern of CsW_2O_6. The yellow and white indices indicate the *hkl* values that satisfy and do not satisfy the $h + l = 4n$ relation (cubic unit cell), respectively. (B) Schematic picture of the atomic displacement that can reproduce the observed diffuse scattering.

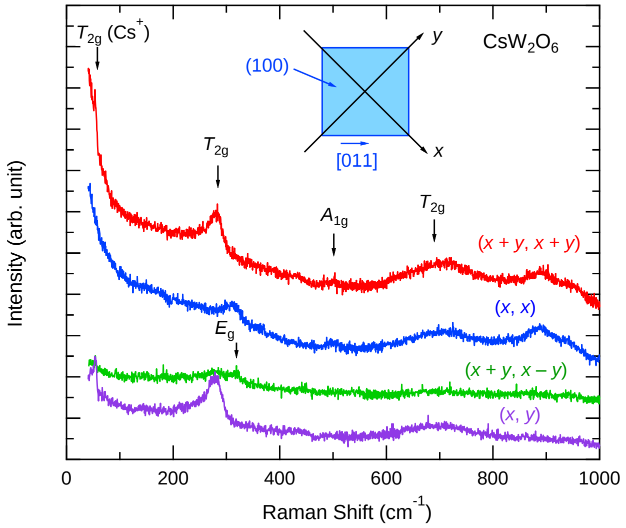

Supplementary Figure 9. Raman spectra of the (100) surface of the CsW_2O_6 single crystal measured at room temperature and a representation of each peak. The polarization directions are described as (x, y), where the x and y vectors in the parentheses are the directions of the electric field of the incident and scattered light, which are indicated in the inset, respectively. The broad peaks above 600 cm^{-1} in the (x, x) and $(x+y, x+y)$ spectra may be considered as two-phonon scattering, although their origin is unclear at present.
